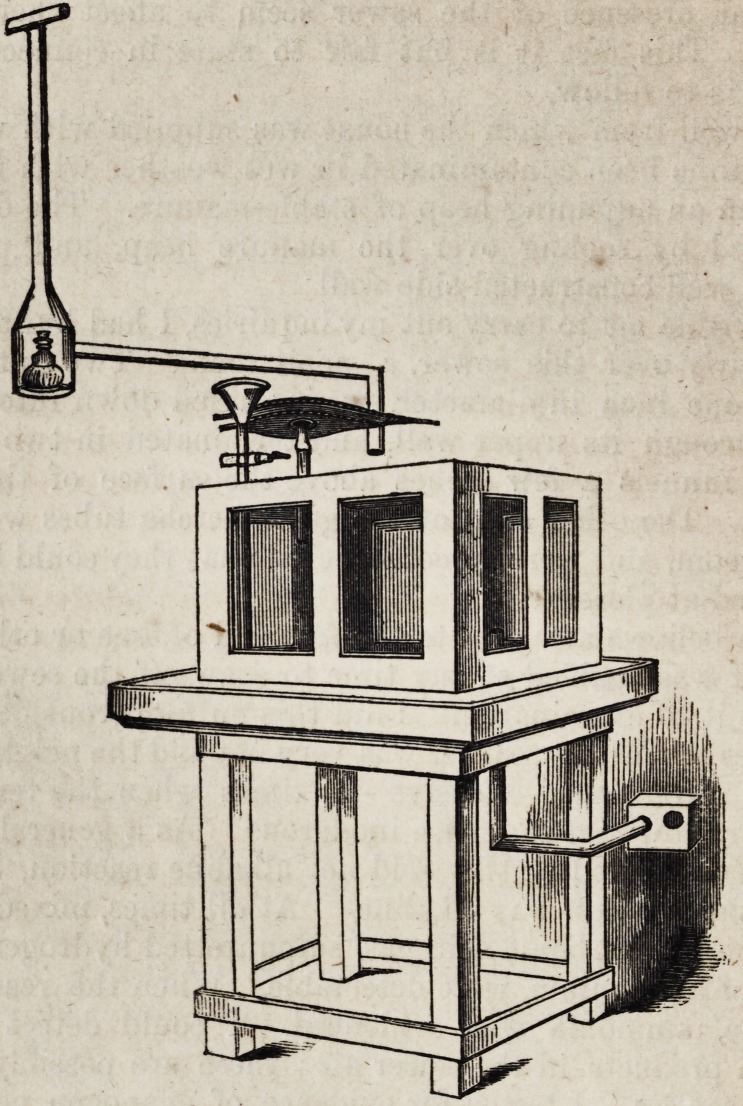# Influence of Sewer Emanations

**Published:** 1858-04

**Authors:** T. Herbert Barker


### THE INFLUENCE OF SEWER EMANATIONS.

By T. HERBERT BARKER, M.D., F.R.C.S.

I HAVE lately been making some inquiries as to the influence on the health of animals, of exposure for a long time to air rendered impure by the diffusion through it of emanations from sewers. The full details of these experiments are recorded elsewhere*; but, as the subject has important sanitary bearings, I send to the SANITARY REVIEW an outline of my researches, that what has been done, small as it is, may become the common property of the profession and the public.

The gaseous emanations from sewers have been subjected, to a certain extent, to chemical analysis. There have been thus detected in them sulphuretted hydrogen gas, sulphide of ammonium, carbonic acid, nitrogen, sometimes phosphuretted hydrogen, and various organic living products. Dr. Odling has recently pointed out the diffusion of an alkaline gas through sewer air. The subject demands much more attentive inquiry than has yet been bestowed on it. Such observations as I have made add but little to what has been previously told by the chemists. A physiological rather than a chemical history is before me.

For the purpose of experiment, I selected a large cesspool, which received, together with the animal excreta, the liquid refuse of an inhabited house. The cesspool was full, and had

* In a MS. essay "On Malaria", written for the Fothergillian Prize of the Medical Society of London for 1858.

at all times so bad a smell, that during hot weather the vicinity was scarcely tolerable. The inhabitants of the house, however, had not for many years suffered from any epidemic; nor did the near presence of the sewer seem to affect their general health. This fact it is but fair to state in connection with what has to follow.

The well from which the house was supplied with water had at one time been contaminated in wet weather with the drainage from an adjoining heap of stable-manure. The defect was remedied by roofing over the manure heap, and protecting it by a well constructed side wall.

To enable me to carry out my inquiries, I had built, close by and nearly over this sewer, a small room. Two gutta percha tubes, one inch in diameter, were carried down into the cesspool through its upper wall, and terminated in two large inverted funnels a few inches above the surface of the sewage matter. The other ends of the gutta percha tubes were in the small room, and were so constructed that they could be opened or closed at pleasure.

By a bellows attached to the free end of one or other of the tubes, I was enabled at any time to draw off the sewer air and subject it to examination. I did this on numerous occasions—at times when the weather was very hot and the neighbourhood of the sewer most offensive—at times when the temperature was very low and the place inodorous. As a general rule, the sewer gas yielded neither acid nor alkaline reaction, but sometimes the reaction was alkaline. At all times, mixed with the common air, carbonic acid gas, sulphuretted hydrogen, or sulphide of ammonium, were detectable. When the reaction was alkaline, ammonia was evidenced. I could detect no other foreign products in the sewer air; these are possibly common to all sewers. I tested for evidence of cyanogen compounds, without any affirmative indication.

When this inquiry had progressed for several weeks, I set to work to try what the influence of sewer air was on animals exposed to it for a long time. For this purpose, I had made a chamber as represented in the drawing. The chamber is an imitation of one constructed, and for many years used in his inquiries, by Dr. Richardson. The chamber which is shown in the drawing subjoined, was made of wood and glass. It had within a cubic measurement of 5,832 cubic inches. In order to keep up a current of sewer air through the chamber, I introduced one of the gutta percha tubes into it at the lower part; from the upper part I carried a tube in the form of a small chimney, as represented in the drawing. At the point where the long tube piping from the chamber makes a right angle upwards, it expanded into a conical box, in which a lamp was placed, so as to create when alight a constant upward draught. The whole played well. When the chamber was closed and the lamp arranged, a current of the sewer air was kept steadily passing through it.

I also attached a pair of bellows to the chamber, in such a way that I could, whenever I was disposed, remove the air by working them, and subject it to investigation, without interfering either with the experiment which might be progressing. In the experiments to be related, the animals operated on were placed in the chamber, were fed by the funnel depicted in the drawing whenever necessary, and were subjected to the sewer gases as is now to be described.

On placing a young dog in the box at twelve o'clock noon,

I kept a current of the cesspool air passing constantly through the chamber by means of the chimney draught. I obtained decided symptoms. Half an hour after the exposure, he became very uneasy and restless; he vomited, and had a distinct rigor. In the course of the day he suffered from diarrhœa and tenesmus. After twelve hours exposure, he was allowed fresh air; but on the next day, when he was removed altogether, he was exhausted. The diarrhœa and vomiting had ceased, but he refused food for some hours. However, he soon recovered.

The air breathed in the chamber by this animal yielded evidence of sulphuretted hydrogen.

On placing another dog in the box connected with the cesspool, and subjecting him to a free current of the foul air, similar results occurred. In ten minutes the creature became very uneasy, and soon afterwards suffered from vomiting and diarrhœa. After these effects, however, he suffered but very little, although kept in the chamber for five hours. After removal, he quickly recovered.

A mouse placed in a cage was let down into the cesspool, to within three inches of the surface of the contained soil. The cesspool was freely open above, so that there was no exclusion of air. The animal was also well plied with food. After this exposure for four days, the animal seemed lively and well, and took his food heartily. On the next day he was found dead.

Another dog was subjected to the cesspool air during a period of twelve days, with such brief intermissions only as sufficed for rapid cleansing of the box. Throughout the time, food was liberally supplied him. The results were as follow:—

During the first day the animal was restless and uneasy, and refused food. On the second day vomiting came on, and was repeated frequently during the day. In the afternoon there was diarrhœa, accompanied by thirst and restlessness. On the third day, in the morning, he had marked shiverings, and refused all food. The feet were somewhat swollen. Towards evening he slept, but had a peculiar kind of tremor with each inspiration. On the fourth day he took food, and drank some milk. He slept during the forenoon, but was restless towards evening. On the fifth and sixth days he was much the same. On the seventh day he was restless and relaxed, and ate no food. On the eighth day he ate but little food, and was restless; he was by this time thinner and feeble. On the ninth day, he had eaten no food for two days, and seemed very ill and miserable. He was therefore taken from the box while it was cleansed, and offered food, which he ate voraciously and to repletion. When removed from the box his skin was preternaturally hot and dry; he was very weak, and his gait feeble. On the tenth day his appetite was better, but he vomited and had diarrhœa in the evening. On the eleventh day he was very restless, and had but little appetite; and on the twelfth, the symptoms being much the same, he was removed to his kennel. He walked feebly; but soon after his liberation ate heartily of food. He continued very thin and weak for six weeks after his removal from the cesspool air.

Having thus ascertained in some measure what was the effect of long exposure to the vitiated air of the cesspool, I instituted another series of experiments. In lieu of exposing animals to this vitiated air, I subjected them, in the same chamber, to certain percentages of such of the individual gases as I had found at various times emanating from the cesspool. By comparing any results that might thus be obtained, with those which had already been obtained, I hoped to obtain a clue to the agent which, in the compound cesspool air, gave rise to the symptoms described.

*Sulphuretted Hydrogen.* I placed a puppy in the box as before, and introduced 100 cubic inches of sulphuretted hydrogen, or 1·714 per cent. The breathing became instantly laboured. In two minutes the animal fell insensible on his side, and in another half minute he was dead without a struggle.

An hour after death, the right side of the heart was found filled with fluid blood to distension. In the left side the blood was partly coagulated. The fluid blood coagulated quickly when received into a glass. The corpuscles of the blood were natural. The lungs were congested in the lower lobes and posteriorly. Above, they were pale and free from congestion. The stomach and abdominal viscera were healthy. The vessels on the surface of the brain were slightly congested.

I placed a puppy in the box as before, and drove in twenty-five cubic inches of sulphuretted hydrogen, or 0·428 per cent. In three minutes the animal fell on his side insensible. In this condition he lay for an hour, without any indication of pain, but with catching respiration. At the end of an hour he ceased to breathe.

Directly after death, the lungs were found generally pale, and were free from congestion. The right side of the heart was filled to distension with blood. The left side contained fluid blood. Blood coagulated in eight minutes after being removed from the body. It was dark in both cavities, and the corpuscles were irregular. They floated about freely between the slips, but not one was natural. Some were crenated at the edges, and thus shrunken and broken up. The stomach presented nothing unnatural. The vessels of the brain were congested.

At thirty-seven minutes past four P.M. a dog was placed in the box, and twelve cubic inches of sulphuretted hydrogen gas, or 0·205 per cent., were slowly introduced. Within a minute, he fell on his side and was seized with tremors. The action of the heart became irregular, and within four minutes the respiration had apparently ceased. This cessation of respiration continued for about two minutes, when he began to breathe heavily. The respiration next became very quick and catching. Afterwards the quick respiration came on in paroxysms, with an occasional long-drawn inspiration. In three quarters of an hour from the commencement, the respirations were 112 per minute, rising sometimes to 120; they then became deeply stertorous, as in apoplexy. I removed this dog from the box at fifteen minutes past six, having exposed him to the gas one hour and thirty-eight minutes. The respirations were at this time stertorous, the limbs were rigid, and the head was drawn backwards. The respiration became gradually more feeble and catching, as if solely diaphragmatic, with a kind of hiccup. The body was universally cold. The respiration then became very peculiar, consisting of two short inspirations to one expiration; and at fifteen minutes past two A.M. the dog died, nine hours and thirty-eight minutes after the commencement of the experiment.

On examination twenty hours after death, there was moderate cadaveric rigidity. The brain was found slightly congested externally, but presented no bloody points. The lungs were collapsed, dark in patches, and congested. The heart was enormously distended, and was remarkable for being excessively loaded with separations of fibrine. The right auricle, pulmonary artery, and the left auricle were literally distended with fibrinous concretions, to the almost entire exclusion of red blood. The right and left ventricles contained a large quantity of dark clotted blood, but there were some separations of fibrine in these cavities also. The fibrinous concretions in the right auricle and pulmonary artery were of pure whiteness. Those on the left side were red and striated, very closely resembling muscular fibre. The liver and spleen were congested. The kidneys were normal. The stomach, viewed externally, had a vascular appearance, but internally, the mucous surface was natural. There was no serous effusion into the abdominal cavity, nor any particular inflation of the alimentary canal with gaseous matters.

Another dog was put into the box, into which there were introduced twelve cubic inches of sulphuretted hydrogen, or 0·205 per cent. He suffered from violent tremors and shortness of breathing. When nearly an hour had elapsed, he appeared better, and was removed at the end of five hours, not labouring under any morbid symptom.

A jackdaw was placed in the chamber, now altogether disconnected from the cesspool. Through the air of the box were diffused nine cubic inches of sulphuretted hydrogen, or 0·154 per cent. Within two minutes the bird essayed to vomit, and almost instantly afterwards was purged. He was incessantly restless, and the breathing was remarkably hurried and catching. After inhaling the gas for ten minutes, his movements became so feeble that it was with difficulty he stood. The pupils, at first contracted, soon became widely dilated. The beak was set widely open; and the tongue, dry and dark at the top, was protruded at each inspiration. After remaining in this condition for an hour and a half, he was removed from the box, and soon recovered.

A dog was placed in the box at eight a.m., and nine cubic inches of sulphuretted hydrogen, or 0·154 per cent., were introduced. Within two minutes the respiration became quickened, with reeling. For a quarter of an hour he was restless, and walked with difficulty. His movements were more like those resulting from intoxication than I had ever seen in a lower animal. This effect gradually subsided; and I took him out of the chamber in three hours, merely enfeebled.

Another dog was placed in the chamber. When he was composed to his new situation, six cubic inches of sulphuretted hydrogen, or 0·102 per cent., were introduced. At first there was watering of the eyes, followed by signs of thirst, muscular debility, and slight drowsiness. In half an hour the breathing had become hurried, and an hour later he suffered from violent diarrhœa; the breathing became more rapid, and the tremors more intense. Three hours after his first introduction the respiration was still hurried, and the heart beat so rapidly that it could not be counted with precision. I calculated, after several attempts to reckon the beats, that there were at least 240 in the minute. He was now again purged. Removed from the chamber, he soon recovered in the pure air.

Another jackdaw was put into the box as before, with six cubic inches of sulphuretted hydrogen, or 0·102 per cent. Within two minutes the bird commenced to vomit, (a curious symptom to observe in birds), and he was also freely purged. These symptoms continued for twenty minutes; afterwards the respiration was very hurried. After keeping him in the box for two hours without much further modification of symptoms, he was removed, and soon recovered.

I put a common hedge-sparrow into the box, as before, with six cubic inches of sulphuretted hydrogen, or 0·102 per cent. Within two minutes he fell down insensible, and continued in this condition for the space of a minute. Respiration next became very hurried and gasping. He rose, but staggered a good deal and fell again on his back. Six minutes after the commencement of the experiment, he vomited, became convulsed, and died in fifteen minutes.

A linnet was placed in the same box as used in the preceding experiment, and without any further introduction of gas. It was put in within ten minutes after the commencement of the preceding experiment. The respiration became hurried at first, but this passed off in the course of half an hour. At noon, *i. e.*, one hour and seven minutes after introduction into the box, I removed it, apparently well, but it died in the evening.

A dog was introduced into the box as before, and three cubic inches of sulphuretted hydrogen, or 0·056 per cent., were driven in. He suffered almost at once from tremors of the muscles. The respiration was also quickened, and the heart-beat was extraordinarily rapid. At the same time he seemed sufficiently lively. After keeping him in the box for two hours, he was let out. The pulsations of the heart could be heard at a short distance from his body, the action was so intense. After removal, he was freely purged for a few hours, but eventually got quite well.

*Sulphide of Ammonium.* From sulphuretted hydrogen I next turned to sulphide of ammonium. This was diffused in vapour from its solution into the chamber in each experiment.

A large dog was placed in the box as before, and six drachms of sulphide of ammonium were introduced. He soon suffered from lacrymation, restlessness and vomiting. The vomited matters gave off copious white fumes. There was a peculiar harsh noise during expiration. In five hours he had recovered, and was then removed.

A dog was placed in the box with half an ounce of sulphide of ammonium. For ten minutes he laboured under excitement with lacrymation. He also had some tremor and tenesmus. The symptoms subsided, and he was removed from the box in five hours.

A jackdaw was placed in the box, and half an ounce of sulphide of ammonium was introduced. The bird vomited, and the vomited matters were of a yellow colour ; the beak was separated ; the tongue was dry and dark-coloured at the top. He was much purged, and the ejected matters were liquid. He expanded both his wings to support his body. The respiration became quicker, and he died in two hours.

After death the blood remained fluid; the lungs were congested; the brain was congested. The other viscera were healthy.

I placed a dog in the box with one ounce of sulphide of ammonium. He soon laboured under profuse lacrymation and salivation, and became very restless. Within five minutes tenesmus showed itself. The respiration became hurried and difficult. He died within ten minutes.

Twenty-four hours after death, the right auricle and ventricle were found filled with quite liquid blood. The left cavities contained a small quantity of fluid blood. The venæ cavæ were distended with fluid blood. Both lungs were deeply congested, and of a dark colour. The vessels of the brain were congested. The stomach was distended with food and an offensive gas. It presented a reddened appearance of the mucous surfaces. The other viscera were of healthy appearance.*

*Carbonic Acid.* A hedgehog was placed in the box, and 88 cubic inches (1½ per cent.) of carbonic acid were introduced. For a quarter of an hour he remained curled up; he then breathed more quickly—sometimes irregularly, and occasionally drew a long inspiration. Soon afterwards he was very restless—running about and trying to escape. He was also freely purged. He became quieter afterwards, and was removed in four hours and a half, upon which he recovered.

I made afterwards several experiments with carbonic acid gas, exposing the animals subjected to experiment to 5, 2½ and 1½ per cent. of that gas. The effects were mainly referrible to impeded respiration, but in one instance diarrhœa was the result.

From the history of these experiments, few as they are, much useful information is attainable. They have brought before us the effects of the compound impure cesspool atmosphere: and they have shown the specific influence of certain particular gaseous poisons, which alone, or in company, emanate from the cesspool, and the decomposing vegetable heap, to pollute filthy localities.

In the first place, it cannot be doubted that cesspool emanations are, when steadily inhaled, poisonous. The dogs subjected to the cesspool air were all affected more or less. The symptoms were those of intestinal derangement followed by

* I have made six other experiments with sulphide of ammonium, but the results are so similar that it would be repetition to record them.

prostration, heat of the surface of the body, distaste for food, and those general signs which mark the milder forms of continued fever common to the dirty and ill-ventilated homes of the lower classes of men.

The peculiar poisonous action of sulphuretted hydrogen is well illustrated in these experiments. It will be observed that the symptoms produced even by the same dose differed in degree in different animals of the same class, the one animal dying from the effects of a dose which was insufficient to do more in the other than produce dangerous symptoms.

The symptoms arising from sulphuretted hydrogen are well marked, and may be considered specific. Vomiting and diarrhœa are the first and most prominent symptoms. The latter is painful; the vomiting is difficult and exhausting, and eventually there is insensibility and entire prostration. When the dose of the poison is at first very large, the prostration and the insensibility are immediate.

The pathology following such poisoning is definite. If the death take place quickly, the pathological evidence is the evidence of asphyxia; if the poison is long breathed in diluted dose, the pathology is modified, the fibrine of the blood is separated, and the heart is slowly clogged up with fibrinous depositions.

The dose of sulphuretted hydrogen required for the production of the specific symptoms is tolerably well shown. It is clear that so little as 0·428 per cent. is a dose absolutely and rapidly poisonous; that so little as 0·205 per cent. may be fatal; and lastly, that so minute a dose as 0·056 per cent. is sufficient to produce serious symptoms, eructations, tremors, rapid and irregular respiration, extraordinary rapidity of the pulse, and diarrhœa.

The effects of sulphide of ammonium, while they differ from those produced by sulphuretted hydrogen, are in themselves sufficiently distinct. Vomiting is a symptom of this poison, without purging, but occasionally with tenesmus. When the dose is very large, death occurs speedily, with quickened and laboured respiration. When the administration is kept up in small doses for many hours, the symptoms are those of excited circulation and thirst, followed by rapid sinking. The surface of the body, from being unusually hot becomes unusually cold. The tongue is protruded, dry, dark, and cold. There is constant jactitation of the limbs, subsultus tendinum, feeble, quick pulse, and ultimately death, which may occur even some hours after the animal has been removed from the poison and placed in the open air.

The pathology after death from sulphide of ammonium differs from that which follows the administration of sulphuretted hydrogen. When the exhalation is prolonged, and the death is gradual, the alimentary mucous surface is changed. The mucous coat is injected and softened in patches. The blood shows no fibrinous separations, but is dark, and either feebly coagulated, or entirely fluid. The blood-corpuscles are also much dissolved and changed, and there is congestion of fluid blood in all the vascular organs.

The dose of sulphide of ammonium required for the production of serious symptoms is difficult to calculate, and this, from the fact, that when the vapour of sulphide is diffused through a confined space, in which an animal is breathing, there is quickly a deposit on the floor of the chamber of the white bicarbonate of ammonia. This deposition is so rapid, indeed, that the effect of the poison is very quickly lost, so that constant renewal is required, and the calculation of dose is necessarily rendered obscure, since the animal is not breathing the same dose for any two minutes together. Dr. Richardson, in his late valuable work on the blood, describes the symptoms resulting from the action of ammonia or its salts as essentially typhoid. My experiments entirely confirm his observations. He remarks as follows:

"We have seen by direct experiment what the effects of ammonia are when it is thrown into the body in large quantities. Thus introduced, it produces what may be unhesitatingly considered *typhoid* symptoms. The tongue becomes dry and dark; there is an involuntary action of the muscles, varying from subsultus to violent convulsions; there are insensibility, extreme sensitiveness to sound, obscurity of sight, and ultimately, if matters are pushed far enough, death by coma. The morbid anatomy is equally demonstrative. The blood is dark and fluid; the serous membranes show petechial spots; the tissues are softened; and, in an experiment which I have lately performed on a dog, sulphide of ammonium being inhaled, there were patches of ulceration extending along the alimentary tract."*

For some months past the medicinal treatment of my fever cases has almost been restricted to small doses of the diluted hydrochloric acid, and the results have been most satisfactory. I notice too, in the Clinical Reports in the *Lancet,* that Dr. Chambers has lately been pursuing a similar plan of treatment, with considerable success. Dr. Richardson's important physiological researches will afford a satisfactory solution.

* Essay on the Cause of the Coagulation of the Blood. By B. W. Richardson, M.D. p. 345.

The symptoms arising from carbonic acid gas have been described so often by various authors, that I need not dwell on them, nor have I pressed them far experimentally. The respiration suffers first from this poison; there is prostration; and if the inhalation is prolonged, diarrhœa. The effects vary with the dose: the instances I have given above are the effects of a small long-continued dose. In larger proportions, insensibility, coma, and asphyxia, are the results.

The pathology varies. While congestion of the lungs is commonly noted as the leading pathological sign, it is clear, from one of my experiments, that when the gas has been long inhaled in small quantities, this rule is not without its exception; for, in one of my cases, the lungs were found of bright vermilion colour, and free from congestion.

The effect of carbonic acid gas on the blood is definite; it does not produce the fibrine deposit like sulphuretted hydrogen, nor the complete fluidity of sulphide of ammonium. But there is feeble coagulation, and sometimes a dark colour even in the arterial blood. If this gas be breathed continuously for a long time in a very minute dose, the brain suffers from congestion of blood, and the mucous membrane of the stomach is injected and reddened.

When the gas has been breathed for a long time in small quantities, so as not to produce insensibility, the effect does not pass off so speedily on placing the animal in the open air as is generally believed. In one of my experiments with carbonic acid, the animal, after being exposed for two hours to an atmosphere in which he breathed from the first two per cent. of carbonic acid, was left (not apparently suffering much) with pure air entering freely into his chamber. Yet he died after all.

The smallest dose of carbonic acid required to produce dangerous symptoms, cannot be determined absolutely from the experiment of placing an animal in a closed chamber and introducing the gas, inasmuch as the gas is also streaming off from the animal itself. I think, however, that the inference is quite fair, that from one to two per cent. of this gas is sufficient, when long inhaled, to produce decided symptoms of imperfect oxidation of the blood, and all the after prostration incident to such interference with the primary act and principle of life.

The symptoms which have thus been noticed as resulting from the inhalation of sulphuretted hydrogen, sulphide of ammonium, and carbonic acid, are sufficient to account for the effects arising from cesspool effluvia, without seeking for any further product from such emanations. Comparing the experiments with cesspool air with those in which separate gases were employed, the inference seems clear to my mind, that the symptoms arising from the inhalation of the cesspool atmosphere were due mainly to the presence of a small amount of sulphuretted hydrogen, which gas was always present. If the experiments with the cesspool air be placed side by side with those in which sulphuretted hydrogen, in the proportion of 0·056 per cent., was administered by inhalation, the analogy between the two sets of results will be sufficiently unmistakeable.

## Figures and Tables

**Figure f1:**